# Endometriosis Nodule Causing Spontaneous Haemoperitoneum in Pregnancy: A Case Report and Literature Review

**DOI:** 10.1155/2017/3480287

**Published:** 2017-04-27

**Authors:** Junaid Rafi, Geetha Mahindrakar, Debjani Mukhopadhyay

**Affiliations:** Ipswich Hospital NHS Trust, Heath Road, Ipswich IP4 5PD, UK

## Abstract

Spontaneous haemoperitoneum in pregnancy (SHiP) due to endometriosis is a very rare condition and this is a case of a 41-year-old primigravida, who presented at 32 weeks with sudden onset of severe lower abdominal pain without any uterine activity. This was a dichorionic-diamniotic twin pregnancy, following in vitro fertilisation for subfertility secondary to severe endometriosis. On admission, pain score was eight, with ten being the maximum of the scale. The vital signs were stable. Abdominal palpation revealed generalised tenderness with no guarding or palpable contraction. There was no evidence of bleeding and the cervical os was closed on speculum examination. The cardiotocograph (CTG) was pathological and a plan was made to deliver the babies with emergency caesarean section. Intraoperatively, there was massive haemoperitoneum which was managed successfully with the involvement of multidisciplinary input from general surgeons and urologists with optimum maternal and fetal outcome.

## 1. Introduction

Spontaneous haemoperitoneum in pregnancy (SHiP) due to endometriosis is very rare. Preoperative diagnosis can be challenging as SHiP may not always present with signs of haemorrhagic shock. We present such a case which was managed successfully in pregnancy with optimum foetal and maternal outcome.

## 2. Case Report

We present the case of a 41-year-old primigravida, who presented at 32 weeks with sudden onset of severe lower abdominal pain without any uterine activity. This was a dichorionic-diamniotic (DCDA) twin pregnancy, following IVF (in vitro fertilisation) for subfertility secondary to severe endometriosis. This was her fourth admission with abdominal pain in pregnancy. On each previous admission, no definite cause was found and a laxative was given to treat constipation.

On admission, pain score was eight, with ten being the maximum of the scale. The vital signs were stable. Abdominal palpation revealed generalised tenderness with no guarding or palpable contraction. There was no evidence of bleeding and the cervical os was closed on speculum examination. The cardiotocograph (CTG) showed unprovoked atypical deceleration with reduced baseline variability which classified CTG as pathological in nonlabouring women. Hence, a plan was made to deliver the babies with emergency caesarean section. Haemoglobin was 105 gm/L, and it was 111 gm/L two weeks prior to this surgery.

Intraoperatively, there was massive haemoperitoneum although the uterus was intact. After delivery of twins and closure of the lower segment, the uterus was exteriorised and the abdominal cavity was explored to find the source of this unexpected bleeding. On elevating the uterus from the pelvis, fresh arterial bleeding was observed from an approximately 4 cm spherical necrotic tissue close to the left uterosacral ligament. This necrotic tissue was sloughed off leaving a cavity, haemostasis was secured by stitching the bleeding point by using a 2/0 PDS suture, and the bleeding was stopped. There was no breach of integrity or communication between the posterior uterine wall and endometrial cavity. As the left ureter was against the left wall of this cavity, the urologist was requested to stent the left ureter prophylactically before further exploration was done. Upper abdominal exploration by general surgeons also revealed an intact liver and spleen and no other cause of bleeding was identified. There was evidence of old haematoma in the Pouch of Douglas (POD) which may suggest gradual smaller bleeds from the necrotic mass correlating with abdominal pain on previous admissions. Total blood loss was 5000 mL intraoperatively. The patient was adequately resuscitated by the anaesthetic team and received six units of packed red blood cells and two units of fresh frozen plasma.

Postoperatively, the patient was transferred to the ITU and made uneventful recovery. She developed postpartum cardiomyopathy two weeks following delivery. Regular follow-up was arranged by the Cardiology Team ten months postnatally; cardiac investigations including echocardiography and cardiac MRI revealed normal cardiac function and structure. Histology of the necrotic tissue confirmed endometriosis.

## 3. Discussion

Spontaneous haemoperitoneum in pregnancy (SHiP) due to endometriosis is a rare condition and was an unexpected finding in our case given the stable observation with no drop in haemoglobin.

The patient was investigated in her previous hospital for infertility and diagnosed to have stage III endometriosis (ASRM classification). She had laparoscopic partial cystectomy done for bilateral ovarian endometriomas and endometriotic spots were found on the sigmoid colon as well. A year later, a second operative laparoscopic diathermy treatment to pelvic endometriotic spots was performed along with dye test revealing bilateral patent tubes. The endometriosis involved the ureters also and an attempt was made to lift the pelvic side wall overlying the ureters; however, the peritoneum was quite thick making endometriotic spot complete excision difficult. In the end, there was a small amount of endometriosis left untreated close to the left ureter. We assume this site may correlate with the same necrotic area found at caesarean section at the back side of the uterus close to the left ureter.

In the literature, the pathophysiology of haemoperitoneum from endometriosis is explained as stimulation of preexisting endometriotic implants by endogenous progesterone inducing decidualisation process [1] and invasion of the adjacent vessels or bleeding from decidualised endometriosis implants [2] resulting in spontaneous bleeding. SHiP can happen in any trimester but most commonly during the third trimester [3]. This condition has been described outside labour in 61%, 18% during labour [4], and 21% in the early postpartum period [5].

SHiP is unpredictable and usually presents with vague abdominal pain in women with a history of endometriosis not relieved by mild analgesia or tocolytics [6].

Endometriosis in pregnancy is associated with perforation of the appendix and sigmoid colon [7, 8], rupture of the uterus [9], and spontaneous bleeding from uteroovarian vessels secondary to endometriosis and urohaemoperitoneum [10].

In the literature, we find conflicting evidence regarding the impact of endometriosis on the pregnancy outcomes.

A big cohort study from Canada [11] looked into the adverse outcomes in 31,068 women who had a pregnancy between 1997 and 2008. They concluded that 784 women had endometriosis, and out of those (*n* = 784) 183 had increased incidence of fetal loss, including spontaneous abortion and stillbirth.

A longitudinal observational study from Japan [12] identified 330 women (out of 9,186 participants) with a history of endometriosis, showing significantly increased incidence of preterm PROM (premature rupture of membranes) and placenta previa regardless of receiving ART (assisted reproductive technology). However, the authors highlighted that the study was based on patient self-reported questionnaires information on whether they had been diagnosed with endometriosis during the past year, ever had endometriosis, or ever received infertility treatment.

In a nationwide Swedish study [13] including 1,442,675 singleton births, researchers assessed the association between adverse pregnancy outcome, ART (assisted reproductive technology), and a previous diagnosis of endometriosis. They found out that endometriosis appears to be a risk factor for preterm birth, caesarean section, antepartum haemorrhage, placental complications, and preeclampsia irrespective of ART used.

On the contrary to the abovementioned studies [11–13], a systemic review [2] concluded that complications of endometriosis during pregnancy are rare and there is no evidence that the disease has a major detrimental effect on pregnancy outcomes.

Similarly, in the context of endometriosis and IVF pregnancies, a recent retrospective case matched study [14] of 239 women concluded that the rate of preterm birth, live birth, incidence of hypertensive disorders, gestational diabetes, small and large for gestational age newborns, and neonatal problems did not differ in IVF treated versus control group; however, placenta previa was more common in women with endometriosis than controls.

It is difficult to predict the pregnancy outcome and complications associated with endometriosis as major studies conducted in the past were based on either personal history of endometriosis given by patients or retrospective case notes review. Hence, not only does extracting the data regarding the staging, severity, and spread of the disease become challenging for researchers, but it is also very difficult to fully appreciate the impact of each specific stage of disease on the pregnancy outcome.

High index of suspicion is required with previous history of endometriosis if women present with irregular uterine contractions and symptoms like severe abdominal pain not relieved by tocolysis with gradually unexplained drop in haemoglobin in the absence of overt haemodynamic instability.

## 4. Conclusion

Antenatal recommendations for specific stage or severity of endometriosis cannot be made; however, it is essential to increase awareness among medical professionals suspecting SHiP in pregnant women presenting with severe constant abdominal pain in the absence of preterm labour. As now even more women with severe endometriosis are achieving successful pregnancy with assisted reproductive technologies, the obstetrician should expect increased frequency of occurrence of such cases in the future.
